# BIS Guided Titration of Sevoflurane in Pediatric Patients Undergoing Elective Surgery: A Randomized Controlled Trial

**DOI:** 10.1111/pan.15057

**Published:** 2025-01-04

**Authors:** T. Wesley Templeton, Gijo Alex, Jean D. Eloy, Lindsay Stollings, Richard J. Ing, Eric C. Cheon, Kumar Belani, Ilan Breskin, Peter S. Sebel, Brad M. Taicher, the BTiger Study Group, Nicholas E. Burjek, Alexandra Coffield, Alissa D. Doll, Lynnette Harris, Michael R. King, Shelly Pecorella, Kiley Poppino, Anusha Samant, Rita Saynhalath, Lily Sykes, Lan Chi Tran, Susan Vishneski

**Affiliations:** ^1^ Department of Anesthesiology Wake Forest University School of Medicine Winston‐Salem North Carolina USA; ^2^ UT Southwestern Medical Center Children's Health Dallas Dallas Texas USA; ^3^ Department of Anesthesiology Rutgers New Jersey Medical School Newark New Jersey USA; ^4^ University of Pittsburgh Medical Center, Children's Hospital of Pittsburgh Pittsburgh Pennsylvania USA; ^5^ Department of Pediatric Anesthesiology Children's Hospital Colorado, University of Colorado, Anschutz Medical Campus Aurora Colorado USA; ^6^ Department of Pediatric Anesthesiology Ann and Robert H Lurie Children's Hospital of Chicago Chicago Illinois USA; ^7^ Northwestern University Feinberg School of Medicine Chicago Illinois USA; ^8^ Department of Anesthesiology M Health Fairview, Masonic Children's Hospital, University of Minnesota Minneapolis Minnesota USA; ^9^ Research and Development Acute Care & Monitoring, Medtronic Jerusalem Israel; ^10^ Department of Anesthesiology Emory University School of Medicine Atlanta Georgia USA; ^11^ Department of Anesthesiology Duke University School of Medicine, Duke University Medical Center Durham North Carolina USA

**Keywords:** Bispectral Index, brain monitoring, depth of anesthesia, inhaled anesthetics, processed EEG

## Abstract

**Background:**

In pediatric patients, the use of processed EEG monitoring may reduce the amount of anesthesia administered while maintaining adequate depth of anesthesia.

**Aims:**

The primary aim of this study was to evaluate whether use of a BIS monitor to guide sevoflurane administration might reduce the average end tidal sevoflurane concentration used in children 4–18 years of age.

**Methods:**

Participants in three age groups (4–8, 9–12, and 13–18 years) were randomized to either the BIS guided group or the control group. Use of sevoflurane as the primary maintenance anesthetic was the only requirement in both arms. In the BIS guided group, sevoflurane was titrated to achieve a target BIS value of 45–60 during the maintenance period. In the control arm, clinicians were blinded to the BIS value. Primary outcome was mean end‐tidal sevoflurane concentration during maintenance phase of anesthesia. Secondary assessments included time to discharge and the readiness and quality of recovery as assessed by the Pediatric Anesthesia Emergence Delirium scale, the modified Aldrete Score, and the Wong–Baker FACES scale. An intention‐to‐treat analysis was used to analyze and compare groups.

**Results:**

A total of 180 participants were randomized. Following randomization, 10 participants did not undergo any study procedures, leaving 84 participants in the BIS guided group and 86 participants in the control group. Across all age groups, the average end‐tidal sevoflurane concentration was less in the BIS guided group compared to control (4–8 years: 2.2% ± 0.3% vs. 2.4% ± 0.4%, −0.3% [−0.4%, −0.1%]; 9–12 years: 1.7% ± 0.5% vs. 2.1% ± 0.6%, −0.4% [−0.7%, −0.1%]; 13–18 years: 1.6% ± 0.4% vs. 1.9% ± 0.5%, −0.3% [−0.5%, −0.1%]). No differences in recovery outcomes between treatment groups were observed.

**Conclusions:**

In pediatric participants, the BIS guided group reported a lower average end‐tidal sevoflurane concentration compared to control, though no significant differences in recovery profile were noted.

**Clinical Implications:**

The Bispectral Index (BIS) is a processed EEG tool that can be used to titrate general anesthesia to achieve desired anesthetic depth. Brain monitoring with BIS resulted in lower average end‐tidal sevoflurane concentrations in children aged 4–18 years undergoing general anesthesia.

**Trial Registration:**

ClinicalTrials.gov identifier: NCT04810481

**Abbreviations**
BIS: Bispectral Index
MAC: minimum alveolar concentration
PACU: post‐anesthesia care unit
PAED: post‐anesthesia emergence delirium

## 1 Introduction

The complex decision‐making process required of pediatric anesthesia providers reflects the complexity of childhood development. Pharmacokinetic profiles may differ markedly based on body size, age, or both, and specific concerns related to excessive drug dosage and neurotoxicity exist given the changing sensitivities of the developing brain [1, 2, 3]. The minimum alveolar concentration (MAC) is used to determine dosage of inhaled anesthetics, which can be adjusted based on age, though other physiological factors including body temperature and genetics have been shown to impact MAC and use of multiple drugs can complicate MAC calculations [4]. Titration of inhaled anesthetics can also be monitored through changes in cardiovascular and behavioral responses. Though MAC and cardiovascular response provide insight into certain aspects of anesthesia (paralysis and analgesia) they may not provide clear insight into hypnotic state, which can lead to inadequate or excessive amounts of delivered anesthesia. Monitoring of EEG signals can provide insight into the hypnotic state and aid in anesthetic dosing to achieve desired depth of anesthesia [5, 6]. The Bispectral Index (BIS) uses processed EEG signals to measure depth of anesthesia and permits anesthesia providers to titrate general anesthesia to achieve desired level of consciousness [7, 8, 9].

Sevoflurane is widely used in pediatric patients for its favorable qualities, including quick induction of and emergence from anesthesia [2, 3]. While negative outcomes associated with high doses of inhalational agents remain somewhat unclear in children, the potential sequelae of faster wake ups and overall decreased anesthetic exposure seem appropriate goals for the clinician. In addition to ensuring appropriate dosage for patient safety and outcomes, optimizing anesthetic gas use is also of interest from environmental and cost perspectives [10]. Therefore, aiming to optimize the amount of sevoflurane administered is sensible from patient care, community, and administrative perspectives.

The primary aim of this study was to investigate the utilization of BIS through comparative evaluations of end‐tidal sevoflurane concentration in children 4–18 years undergoing general anesthesia and surgery. The primary outcome was mean end‐tidal sevoflurane concentration. We hypothesized that mean end‐tidal sevoflurane concentration would be lower in participants in the BIS guided group compared to participants in the control group, in which the clinician was blinded to the BIS monitor. The secondary aim was to evaluate differences in the quality and duration of recovery between the two treatment groups. Secondary endpoints included time to discharge from recovery unit and scores on the following assessments: Pediatric Anesthesia Emergence Delirium scale, the modified Aldrete Score, and the Wong–Baker FACES scale.

## 2 Methods

This prospective, multicenter randomized controlled trial was conducted in accordance with the Declaration of Helsinki and all local regulatory requirements. The Bispectral Index and End‐Tidal Anesthetic Gas Concentration in Pediatric Patients undergoing Sevoflurane Anesthesia (BTiger) Trial was registered prior to patient enrollment at clinicaltrials.gov (Registration number: NCT04810481, Date of Registration: March 2021). This study was sponsored and funded by Medtronic, the manufacturer of the BIS device. The trial was conducted between July 2021 and October 2022 at eight sites in the United States. The trial was stopped when the minimum sample size for each age group was achieved. This research was prospectively reviewed and approved by a duly constituted ethics committee at each site. The protocol was approved at each site by an Institutional Review Board (IRB), with associated approval information presented in Table S1. Written informed consent from parent(s) or legal guardian(s) was obtained for all participants under age 18 years. Written assent was obtained for all participants meeting appropriate age criteria and written informed consent was obtained for all participants aged 18 years. All consent and assent were obtained prior to study procedures. This manuscript adheres to the applicable CONSORT guidelines [11].

### 2.1 Participants

Patients 4–18 years were deemed eligible for study participation if they were undergoing routine general anesthesia with sevoflurane, had an expected surgical procedure duration of at least 30 min, and had an American Society of Anesthesiologists physical status score of I, II, or III. Individuals were excluded if they had any of the following: (1) severe contact allergy to standard adhesive materials found in pulse oximetry sensors, ECG electrodes, respiratory monitor electrodes, or other medical sensors, (2) known neurological disorder, (3) currently taking psychoactive medications or medications that may impact the central nervous system, (5) severe developmental delay per assessment of investigator and/or report of parent/guardian, (6) airway abnormalities, (7) pregnancy, (8) planned use of any regional anesthesia or planned use of dexmedetomidine, or (9) interference of study procedures with patient care. Participants were divided into the following age‐based groups: 4–8, 9–12, and 13–18 years.

### 2.2 Primary Outcome

The primary outcome was mean end‐tidal sevoflurane concentration during the maintenance phase of anesthesia in participants using a BIS guided anesthetic protocol compared to control, in which the clinician was blinded to the BIS monitor. The start of the maintenance phase of anesthesia for this study was defined as 10 min after induction and the end of the maintenance phase was defined as the onset of a continuous decrease in sevoflurane administration.

### 2.3 Secondary Outcomes

Secondary outcomes included an assessment of the quality of emergence and timing of recovery following anesthesia in both study groups. Specific measures included: (1) time to post anesthesia care unit (PACU) discharge readiness, (2) modified Aldrete Score (3) Pediatric Anesthesia Emergence Delirium Score (PAED), and (4) Wong–Baker FACES Pain Rating Score. Assessments were performed at 15 min intervals following arrival in the PACU. Other secondary outcomes included the percentage of time during maintenance of anesthesia with the BIS value within target ranges (BIS value: 45–60), and the number of episodes and duration of burst suppression. All adverse events were recorded.

### 2.4 Study Procedures and Anesthesia

Prior to full site activation, each site completed 2–4 training cases, to ensure data quality. Training cases were not randomized and were not included in the final analysis. Once training cases were complete at the individual site, participants were randomized into the two treatment groups. Randomization schedules were prepared per site (eight total schedules) and were assigned sequentially upon site activation. Randomization schedules were generated by the sponsor using SAS statistical software (Version 9.4, SAS Inc., Cary, NC) and were provided to each site via an Excel spreadsheet (Microsoft Corporation, Redmond, WA). Participants were stratified by age group and then randomized using a 1:1 randomization scheme. To ensure adequate sample size in the BIS guided group was obtained for the pre‐planned analysis, data was reviewed throughout the study and enrollment was continued until the required number of participants in the BIS guided group met the target in‐range percentage (65% of maintenance phase with BIS value 45–60). Due to the obvious differences in study procedures between the BIS guided and control groups, the study was unblinded. Research staff at each site enrolled participants and assigned participants to the assigned intervention.

Following enrollment, vital signs were recorded for all participants in the preoperative holding area, regardless of treatment group. Inhalational inductions consisted of sevoflurane alone or in combination with nitrous oxide (N_2_O) in 100% oxygen, with additional intravenous propofol utilized in some cases. Participants with an IV present prior to induction underwent induction with propofol at the discretion of the clinician caring for the patient. Upon arrival to the operating room, another set of vital signs was recorded. According to participant size, a BIS Pediatric sensor or an adult sized BIS QUATRO sensor (Medtronic, Boulder, CO) was placed on the participant's forehead prior to induction, according to the manufacturer's recommendations, though in some cases it was necessary to place the BIS sensor after induction. Once attached to the forehead, the sensor was connected to a sponsor provided BIS Complete Monitoring System (Medtronic, Boulder, CO) and an assessment of signal quality and sensor placement was performed. Additionally, a separate pulse oximeter (Nellcor[TM] N‐600x, Medtronic, Boulder, CO) attached to a study provided laptop was placed on the participant. All devices, including the BIS Complete Monitoring System, pulse oximeter, and the electronic health record were synchronized manually. BIS and pulse oximeter data were recorded continuously. End‐tidal sevoflurane concentration, oxygen, and N_2_O concentrations were recorded by trained, dedicated research staff present in the operating room at 5 min intervals throughout the procedure. Additionally, the timing and nature of any adjustment of the inhaled sevoflurane concentration, along with timing and dosing of all intraoperative medications were recorded throughout the procedure. Airway management approach (endotracheal tube or supraglottic airway) was at the discretion of the clinician.

In the BIS guided group, inhaled sevoflurane was titrated to maintain a BIS value between 45 and 60 during the maintenance period of anesthesia. All adjustments to the inhaled concentration of sevoflurane to maintain the BIS within range were at the discretion of the clinician. For the control condition, anesthetic care including titrations of sevoflurane was provided according to the clinician's expertise and preference. No specific instructions or training were provided beyond the inclusion/exclusion criteria to define anesthetic practice for the control group. The clinician adjusted sevoflurane according to their clinical judgment. Under the control condition, the clinician was blinded to the BIS value using a thick plastic sheet, leaving only the Signal‐Quality‐Index (SQI) number visible to ensure a good quality signal was collected throughout the procedure.

Following closure and at the point of emergence and de‐instrumentation of the airway, participants were observed, and any perioperative respiratory adverse events, including the presence of coughing, choking, desaturation, and laryngospasm, were recorded. Participants were then transported to the PACU.

Once in the PACU, all participants were evaluated using the following assessment tools: PAED, modified Aldrete Score, and Wong–Baker FACES Pain Scale (when awake). These assessments were made on arrival in the PACU and every 15 min thereafter until the patient was determined to be fit for discharge from the PACU. Participants were considered to have completed all study procedures following discharge from the PACU.

### 2.5 Sample Size

Based on published data for ages 3–18 years [12], the maintenance phase sevoflurane concentrations used for the sample size calculation were 1.8 ± 0.4 and 2.4 ± 0.6 for BIS guided and control, respectively. A two‐sample *t*‐test with two‐sided significance level of 0.05 was used to estimate the sample size needed to achieve at least 80% power to detect a 0.5 difference with pooled standard deviation of 0.5 in sevoflurane concentration (%) between BIS guided and control groups. Allowing for 5% drop‐out, it was determined that a minimum of 18 participants per treatment group at each age group was necessary. Thus, to compare treatments between the three age groups [4, 5, 6, 7, 8, 9, 10, 11, 12, 13, 14, 15, 16, 17, 18] a sample size of at least 108 participants was determined.

### 2.6 Statistical Analysis

Two sets of analyses were completed for this study: pre‐planned and post hoc. The pre‐planned analysis was defined prior to study start and only included participants who were compliant with all aspects of the protocol, including staying in the target range (BIS value: 45–60) for at least 65% of the maintenance phase for the BIS guided group. After review of study results by the Food and Drug Administration and peer‐review of the manuscript, a post hoc intention‐to‐treat analysis was completed to include all participants with data. The outcome variables for both analyses remained the same. Results from the pre‐planned analysis are available on clinicaltrials.gov, and for the primary outcome, in Table S2. Given the study design, results from the post hoc intention‐to‐treat analysis are reported in this manuscript.

Following the analysis plan, continuous variables were summarized using descriptive statistics, including mean and standard deviation. Categorical variables are represented as frequencies and percentages. Normality of data was tested using the Kolmogorov–Smirnov test. For continuous variables, a two‐sided *t*‐test or Wilcoxon rank‐sum test, as appropriate, was used to compare the two treatment groups. Categorical variables were evaluated using the Chi‐squared test or Fisher's exact test, as appropriate. Statistical significance was accepted at α < 0.05. Poolability of site data was assessed using a logistic regression model to test for a site effect and significance was accepted at α = 0.15. All analyses were performed using SAS. Post hoc analyses included the intention‐to‐treat analysis that included all randomized participants with data, regression analysis of end‐tidal sevoflurane concentration and a comparison of peak values during emergence for PAED, modified Aldrete score, and Wong–Baker FACES Pain Scale for all three age groups, incidence of emergence delirium, and discharge readiness based on cut‐off thresholds for modified Aldrete score.

## 3 Results

### 3.1 Participants

**FIGURE 1:**
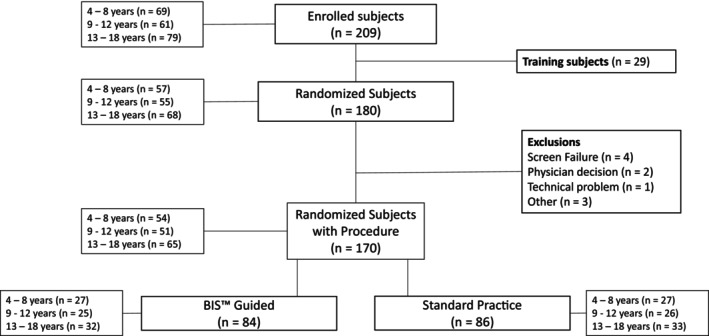
CONSORT flow chart of study enrollment and randomization.

From July 2021 to October 2022, a total of 209 participants were consented, and assented where appropriate, for the study. Of those, 29 participants were training cases who did not undergo randomization. Ten participants were randomized but did not undergo study procedures for the following reasons: four due to screen failure (one participant did not meet all inclusion criteria; three participants met at least one exclusion criteria), two due to scheduling changes, one due to physician decision, one due to technical difficulty, and one due to double enrollment. A total of 170 participants were randomized and underwent study procedures (4–8 years: *n* = 54, 9–12 years: *n* = 51, 13–18 years: *n* = 65). Four participants experienced randomization errors, three participants were randomized to the BIS guided arm but did not receive BIS (all 13–18 years) and one participant was randomized to the control group but did receive BIS (9–12 years). These randomization errors may have most impacted final outcomes in the 13–18 years age group. The flow of participants is summarized in Figure 1.

### 3.2 Demographics

Baseline demographic information is summarized in Table 1. There were no clinically meaningful differences noted in patient demographics or baseline characteristics between the BIS guided and control groups. Overall, procedure time, defined as time from induction to end of procedure, was comparable between treatment arms with a procedure time of 82.3 ± 51.0 min in the BIS guided and 74.8 ± 34.8 min in the control group (BIS‐Control [95% CI]: 7.5 [−5.7, 20.7]). Additionally, there were no statistically significant differences noted in the duration of the maintenance phase between the BIS guided group and the control group, (67.9 ± 46.1 min versus 62.0 ± 31.3 min, respectively, (5.9 [−6.0, 17.8])). Anesthetic mean dosages of concomitant medications including midazolam, adjuvants, analgesics, muscle relaxants, and reversal agents are summarized in Table 2. There were no statistically significant differences in mean dosage for any of the concomitant medications administered during study procedures between the two groups.

**TABLE 1:** Summary of participant characteristics.

Variable	BIS guided (*n* = 84)	Control (*n* = 86)
Age (years)	11.2 ± 4.1 (84) (4–18)	11.3 ± 3.9 (86) (4–18)
Weight (kg)	46.82 ± 23.15 (84) (16.4–121)	48.63 ± 29.15 (86) (15.7–209.5)
Sex		
Female	46.4% (39/84)	40.7% (35/86)
Male	53.6% (45/84)	59.3% (51/86)
Race		
American Indian or Alaska Native	0.0% (0/84)	1.2% (1/86)
Asian	1.2% (1/84)	2.3% (2/86)
Black Or African American	21.4% (18/84)	25.6% (22/86)
White	63.1% (53/84)	59.3% (51/86)
Other	4.8% (4/84)	4.7% (4/86)
Unknown	8.3% (7/84)	7.0% (6/86)
Not reported	1.2% (1/84)	0.0% (0/86)
Ethnicity		
Hispanic Or Latino	25.0% (21/84)	22.1% (19/86)
Not Hispanic or Latino	72.6% (61/84)	74.4% (64/86)
Not reported	1.2% (1/84)	0.0% (0/86)
Unknown	1.2% (1/84)	3.5% (3/86)
ASA physical status classification		
Class I	53.6% (45/84)	47.7% (41/86)
Class II	44.0% (37/84)	47.7% (41/86)
Class III	2.4% (2/84)	4.7% (4/86)
Surgical type		
Abdominal	39.3% (33/84)	40.7% (35/86)
Dental	9.5% (8/84)	4.7% (4/86)
General	1.2% (1/84)	0.0% (0/86)
Ophthalmology	6.0% (5/84)	2.3% (2/86)
Orthopedic	23.8% (20/84)	27.9% (24/86)
Urological	10.7% (9/84)	14.0% (12/86)

*Note*: Age and weight are represented as mean ± SD (*n*), (min, max). Sex, race, ethnicity, ASA physical status classification, and surgical type are represented as % (count).

**TABLE 2:** Anesthetic concomitant medication dosage by treatment arm.

Medication	BIS guided (*N* = 84)	Control (*N* = 86)	*p*
Propofol (mg/kg)	2.4 ± 1.2 (72) (0.1, 5.9)	2.7 ± 1.2 (70) (0.9, 6.3)	0.13
Rocuronium (mg/kg)	0.8 ± 0.3 (56) (0.2, 1.4)	0.8 ± 0.4 (48) (0.1, 2.3)	0.92
Fentanyl (μg/kg)	2.0 ± 1.2 (66) (0.3, 6.8)	1.8 ± 0.8 (67) (0.4, 4.2)	0.30
Acetaminophen (mg/kg)	13.7 ± 3.6 (31) (0.0, 19.0)	13.4 ± 3.8 (23) (4.8, 19.6)	0.79
Ketorolac (mg/kg)	0.4 ± 0.1 (35) (0.2, 0.6)	0.4 ± 0.1 (29) (0.1, 0.6)	0.44
Midazolam (mg/kg)	0.1 ± 0.1 (29) (0.0, 0.5)	0.1 ± 0.1 (38) (0.0, 0.5)	0.90
Sugammadex (mg/kg)	2.8 ± 0.9 (33) (1.8, 4.9)	2.7 ± 1.2 (29) (0.9, 6.4)	0.82
Ondansetron (mg/kg)	0.1 ± 0.0 (59) (0.0, 0.2)	0.1 ± 0.0 (50) (0.0, 0.2)	0.31
Dexamethasone (mg/kg)	0.1 ± 0.1 (49) (0.0, 0.4)	0.1 ± 0.1 (56) (0.0, 0.4)	0.35

*Note*: Continuous variables were assessed using two‐sided *t*‐test. All data represented as mean ± SD (*n*), (min, max).

### 3.3 Primary Outcome

For each age group the mean end‐tidal sevoflurane concentration during the maintenance phase was significantly lower in the BIS guided group compared to control for both the post hoc intention‐to‐treat (Table 3) and planned (Table S2) analyses. A post hoc regression analysis of end‐tidal sevoflurane concentration by treatment arm and age group is presented in Figure S1.

**TABLE 3:** Comparison of end‐tidal Sevoflurane concentration during maintenance phase by treatment arm (intention‐to‐treat, *N* = 170).

Age Group	Statistic	BIS guided (*n* = 84)	Control (*n* = 86)	Mean difference	*p*
4–8 years	Mean ± SD (*n*) 95% CI	2.2 ± 0.3 (27) 2.1, 2.3	2.4 ± 0.4 (27) 2.3, 2.6	−0.3 ± 0.3 (54) −0.4, −0.1	0.01
9–12 years	Mean ± SD (*n*) 95% CI	1.7 ± 0.5 (25) 1.5, 1.9	2.1 ± 0.6 (26) 1.9, 2.4	−0.4 ± 0.6 (51) −0.7, −0.1	0.01
13–18 years	Mean ± SD (*n*) 95% CI	1.6 ± 0.4 (32) 1.4, 1.7	1.9 ± 0.5 (33) 1.7, 2.0	−0.3 ± 0.4 (65) −0.5, −0.1	0.01
All	Mean ± SD (*n*) 95% CI	1.8 ± 0.5 (84) 1.7, 1.9	2.1 ± 0.5 (86) 2.0, 2.2	−0.3 ± 0.5 (170) −0.5, −0.2	< 0.01

*Note*: Significance level set at *p* < 0.05.

Abbreviations: CI, confidence interval; SD, standard deviation.

### 3.4 Secondary Outcomes

Post hoc analyses from the intention‐to‐treat cohort are presented for all secondary outcomes. The mean time to PACU discharge, defined as PACU admission to PACU discharge, for the BIS guided treatment arm was 48.6 ± 26.4 min and 55.7 ± 34.1 min for the control (−7.0 [−16.3, 2.2]). An average of the most severe PAED score, modified Aldrete total score, and Wong–Baker FACES scale assessment for each patient in a given treatment arm are presented in Table 4. Interestingly, the 13–18 year old cohort showed a statistically significant difference in the most severe PAED score between the two group, with the BIS guided group recording a lower peak score than the control group (BIS‐Control: −1.7 [−3.3, 0.0]). A post hoc analysis using PAED total score to compare incidence of emergence delirium between treatment groups was completed, with those reporting a PAED total score > 10 considered to have emergence delirium [5]. Overall, those in the BIS guided group had a statistically lower incidence of emergence delirium compared to the control group (40% versus 60%, respectively, *p* = 0.015). No statistically significant differences in incidence of emergence delirium between groups were noted for any of the age groups. Additionally, using Fisher's Exact Test, a post hoc analysis of most severe modified Aldrete total score prior to discharge, where ≥ 9 was considered ready for PACU discharge [13], found that, overall, those in the BIS guided group recorded scores ≥ 9 more often than the control group (58% versus 42%, respectively, *p* = 0.03), suggesting that those in the BIS guided group showed more signs of discharge readiness during their PACU stay. Further, there was a statistically significant difference in modified Aldrete total score between the two treatment arms overall (BIS‐Control: 0.6 [0.0, 1.3]) and for the 13–18 years age group (BIS‐Control: 1.1 [0.0, 2.2]). Using Fisher's Exact Test, no statistically significant differences between the two treatment arms in Wong–Baker FACES scale were observed in any age group or overall (BIS‐Control: 0.6 [−0.3, 1.5]). Further, a post hoc regression analysis of most severe PAED score and Wong–Baker FACES score was completed to determine correlation between the signs of emergence delirium and reported pain. No correlation between the two scores was observed (correlation coefficient: 0.002). Not surprisingly, there were no statistical differences in PAED total score, modified Aldrete score, or Wong–Baker FACES scale assessment outcomes between treatment groups at final assessment, supporting that participants were appropriately discharged.

**TABLE 4:** Emergence assessment outcomes by treatment group–most severe rating.

Variable	BIS guided (*N* = 84)	Control (*N* = 86)	*p*
4–8 years			
PAED Total score	10.0 ± 3.7 (23) (1.0, 15.0)	10.9 ± 2.8 (21) (1.0, 13.0)	0.40
Modified Aldrete Total score	7.5 ± 2.4 (27) (3.0, 10.0)	7.4 ± 2.1 (27) (4.0, 10.0)	0.63
PAED Total score > 10	60.9% (14/23)	81.0% (17/21)	0.19^a^
Modified Aldrete Total score ≥ 9	51.9% (14/27)	40.7% (11/27)	0.59^a^
Wong–Baker FACES Scale			0.16^a^
No hurt	16.7% (4/24)	45.8% (11/24)	
Hurt little bit	33.3% (8/24)	8.3% (2/24)	
Hurt little more	33.3% (8/24)	29.2% (7/24)	
Hurt even more	4.2% (1/24)	4.2% (1/24)	
Hurt whole lot	8.3% (2/24)	8.3% (2/24)	
Hurt worst	4.2% (1/24)	4.2% (1/24)	
9–12 years			
PAED Total score	9.6 ± 3.7 (18) (1.0, 14.0)	9.7 ± 3.8 (24) (1.0, 14.0)	0.90
Modified Aldrete Total score	8.5 ± 2.0 (24) (3.0, 10.0)	7.9 ± 2.1 (26) (3.0, 10.0)	0.26
PAED Total score > 10	50.0% (9/18)	66.7% (16/24)	0.35^a^
Modified Aldrete Total score ≥ 9	66.7% (16/24)	46.2% (12/26)	0.17^a^
Wong–Baker FACES Scale			0.78^a^
No hurt	8.7% (2/23)	15.4% (4/26)	
Hurt little bit	17.4% (4/23)	15.4% (4/26)	
Hurt little more	34.8% (8/23)	34.6% (9/26)	
Hurt even more	13.0% (3/23)	23.1% (6/26)	
Hurt whole lot	17.4% (4/23)	7.7% (2/26)	
Hurt worst	8.7% (2/23)	3.8% (1/26)	
13–18 years			
PAED Total score	10.1 ± 3.5 (23) (1.0, 14.0)	11.7 ± 2.3 (27) (4.0, 16.0)	0.06
Modified Aldrete Total score	7.9 ± 2.1 (32) (3.0, 10.0)	6.8 ± 2.3 (33) (3.0, 10.0)	0.05
PAED Total score > 10	65.2% (15/23)	88.9% (24/27)	0.08^a^
Modified Aldrete Total score ≥ 9	53.1% (17/32)	33.3% (11/33)	0.14^a^
Wong–Baker FACES Scale			0.60^a^
No hurt	15.6% (5/32)	22.6% (7/31)	
Hurt little bit	31.3% (10/32)	32.3% (10/31)	
Hurt little more	25.0% (8/32)	16.1% (5/31)	
Hurt even more	9.4% (3/32)	19.4% (6/31)	
Hurt whole lot	12.5% (4/32)	3.2% (1/31)	
Hurt worst	6.3% (2/32)	6.5% (2/31)	
All			
PAED Total score	9.9 ± 3.6 (64) (1.0, 14.0)	10.8 ± 3.1 (72) (1.0, 16.0)	0.12
Modified Aldrete Total score	8.0 ± 2.2 (83) (3.0, 10.0)	7.3 ± 2.2 (86) (3.0, 10.0)	0.04
PAED Total score > 10	40.0% (38/64)	60.0% (57/72)	0.02^a^
Modified Aldrete Total score ≥ 9	58.0% (47/83)	42.0% (34/86)	0.03^a^
Wong–Baker FACES Scale			0.14^a^
No hurt	13.9% (11/79)	27.2% (22/81)	
Hurt little bit	27.9% (22/79)	19.8% (16/81)	
Hurt little more	30.4% (24/79)	25.6% (21/81)	
Hurt even more	8.9% (7/79)	16.1% (13/81)	
Hurt whole lot	12.7% (10/79)	6.2% (5/81)	
Hurt worst	6.3% (5/79)	4.9% (4/81)	

*Note*: Significance level set at *p* < 0.05. PAED total score and modified Aldrete total score are represented as mean ± SD (*n*), (min, max). PAED total score > 10, Modified Aldrete total score > 9, and Wong‐Baker FACES Scale are represented as % (count).

Abbreviation: PAED, pediatric anesthesia emergence delirium.

^a^ Fisher's exact test used due to small group sample size.

Across all ages, the percentage of time within the target BIS range of 45–60 during maintenance of anesthesia was 67.8% ± 20.2% in the BIS guided treatment group and 26.0% ± 25.9% in the control group. Ten participants in the BIS guided group experienced burst suppression, with three having episodes greater than 60 s. Of those three participants, all suppression events occurred within the first 8 min of the maintenance phase and their induction included a propofol bolus. In the control group, 15 participants experienced burst suppression, but only seven of the episodes lasted greater than 60 s. Of those seven participants, five participants had suppression events that occurred within the first 15 min of the maintenance phase and all included propofol bolus for their induction. Suppression events that occurred later during the maintenance phase lasted only a few seconds.

### 3.5 Adverse Events

A total of 123 events were reported in the BIS guided treatment arm, compared to 137 events in the control group. These are summarized in Table 5. Overall, the most common events reported in the two treatment arms were tachycardia and hypotension during recovery. Bradycardia was reported fewer times in the BIS group (8.3%, 7/84) compared to control (22.1%, 19/86). No significant morbidity or mortality event was observed in either group. The only perioperative respiratory adverse event noted in either group was “coughing,” which was not severe. There were no instances of laryngospasm or bronchospasm. There was a single instance of desaturation in the BIS guided group.

**TABLE 5:** Summary of perioperative adverse events.

Adverse event type	BIS guided, (*n* = 84)	Control, (*n* = 86)
Movement during the procedure	13.1% (11/84)	10.4% (9/86)
Hypotension during the procedure	0.0% (0/84)	1.2% (1/86)
Hypertension during the procedure	0.0% (0/84)	1.2% (1/86)
Tachycardia in recovery	53.6% (45/84)	40.7% (35/86)
Bradycardia in recovery	8.3% (7/84)	22.1% (19/86)
Hypotension in recovery	38.1% (32/84)	51.2% (44/86)
Hypertension in recovery	32.1% (27/84)	32.6% (28/86)
Oxygen desaturation in recovery	1.2% (1/84)	0.0% (0/86)

*Note*: All data presented as % (count).

## 4 Discussion

The primary finding of this randomized controlled trial was that the use of BIS monitoring to guide the administration of sevoflurane in pediatric participants, aged 4–18 years, reduced the average end‐tidal sevoflurane concentration approximately 14% compared to control. Additionally, the end‐tidal sevoflurane concentration required to maintain the BIS value between 45 and 60 during the maintenance phase of anesthesia appeared to decrease with age. These findings are clinically important because use of the BIS to titrate sevoflurane to achieve an appropriate pre‐determined depth of anesthesia in pediatric patients may allow the clinician to reduce total exposure to inhalational anesthesia.

A statistically lower incidence of emergence delirium (PAED > 10) was observed overall in the BIS guided group, supporting some previous findings that EEG‐guided anesthesia may positively impact patient recovery [5, 14]. Additionally, although we did not see differences in discharge times between the two groups, there was an observed difference in modified Aldrete score between the two treatment arms overall, suggesting that even at their nadir, those in the BIS guided treatment arm showed more signs of discharge readiness than those in the control group.

Previous studies have had mixed results in attempting to demonstrate a clinically relevant difference in the consumption of various anesthetics as well as the potential applicability of the BIS monitor in younger age groups [9, 12, 15, 16]. Differing results in the literature may be related to the primary anesthetic agent being titrated to a given BIS value, as anesthetics differ in their EEG profiles [12, 17]. Consistent with our findings, Bannister et al. found that in children 3–18 years of age undergoing tonsillectomy and adenoidectomy, BIS guided sevoflurane administration significantly reduced the end‐tidal sevoflurane concentration compared to the control. In contrast to our results, they observed significantly less time to PACU discharge in the BIS guided group, compared to control. This difference in findings may have been due to smaller variance in discharge times in the Bannister study, which was completed at one site, compared to this multi‐center study, where slight differences in discharge procedures may have contributed to greater variance. Finally, it is worth noting that BIS monitoring has been noted to lack reliability in infants and toddlers, likely a result of underlying neurodevelopmental differences [15, 18, 19]. The youngest participants included in this study were 4 years of age, thus avoiding these differences.

Though several clinical outcomes were assessed as part of this study, one notable limitation was the lack of formal assessment for intraoperative awareness. Additionally, though the multi‐center design is considered a strength of the study in terms of generalizability, the lack of instruction and/or variability in anesthetic practice employed during the control cases limits the ability to provide a comprehensive explanation of all specific anesthetic approaches used. The lack of a standardized anesthetic regimen beyond the use of sevoflurane and the wide variety of procedure types included may have generated potential, unknown confounding factors that may have impacted results but were not accounted for in the analysis. It is also worth noting that for the BIS guided group, the adherence to the target BIS value range of 45–60 during the maintenance period of anesthesia was achieved, on average across all age groups, 67.8% of the time. It is unclear what factors influenced this adherence rate and future studies may be informative in elucidating patient and/or clinical practice variables that may affect BIS guided anesthetic titration. Finally, post anesthesia care unit assessments were performed by an unblinded observer who was aware of the group assignment which may have influenced their rating of postoperative behavior.

In conclusion, the use of the BIS monitor reduced the amount of sevoflurane administered, although the recovery profile of both groups was similar. Furthermore, the end‐tidal sevoflurane concentration required to maintain the BIS value in an appropriate range during general anesthesia appeared to decrease with age. Further prospective studies using the BIS monitor to guide sevoflurane administration during a standardized anesthetic protocol across a more homogeneous group of surgical procedures should be performed to determine if specific recovery indices might be improved when using the BIS monitor to guide anesthetic administration in children.

## Author Contributions

**T. Wesley Templeton:** helped with data collection, interpretation of results, drafting and editing of manuscript, final manuscript approval. **Gijo Alex:** helped with data collection, editing of manuscript, final manuscript approval. **Jean D. Eloy:** helped with data collection, editing of manuscript, final manuscript approval. **Lindsay Stollings‐Cody:** helped with data collection, editing of manuscript, final manuscript approval. **Richard J. Ing:** helped with data collection, editing of manuscript, final manuscript approval. **Eric C. Cheon:** helped with data collection, editing of manuscript, final manuscript approval. **Kumar Belani:** helped with data collection, editing of manuscript, final manuscript approval. **Ilan Breskin:** helped with study design, data analysis, interpretation of results, editing of manuscript, final manuscript approval. **Peter S. Sebel:** helped with study design, interpretation of results, editing of manuscript, final manuscript approval. **Brad M. Taicher:** helped with data collection, interpretation of results, drafting and editing of manuscript, final manuscript approval.

## Disclosure

This study was sponsored and funded by Medtronic.

## Conflicts of Interest

T.W.T., G.A., J.D.E., L.S., R.J.I., E.C.C., K.B., and B.M.T. (or their institutions) received research support from Medtronic to conduct this study. I.B. is a full‐time employee of Medtronic. P.S.S. is a paid consultant to Medtronic.

## Supporting information


Table S1.

Table S2.

Figure S1.

## Data Availability

The data that support the findings of this study are available from the study sponsor upon reasonable request.
