# Supplementary material for: BIS Guided Titration of Sevoflurane in Pediatric Patients Undergoing Elective Surgery: A Randomized Controlled Trial
Source: Paediatr Anaesth. 2025 Jan 4;35(4):277–86. doi: 10.1111/pan.15057 (PMC11883502; doi:10.1111/pan.15057)
Supplement: Supplementary file 1 — Table S1. [file PAN-35-277-s003.docx]

**Supplementary Tables**

Supplementary Table 1: Site Information

| **Site Number** | **Site Name** | **Institutional Review Board** | **Participants Enrolled** |
| --- | --- | --- | --- |
| 1 | Duke Children’s Hospital and Health Center | WCG | 18 |
| 2 | University of Pittsburgh School of Medicine, Children’s Hospital of Pittsburgh | WCG | 27 |
| 3 | Children’s Hospital Colorado, University of Colorado, Anschutz Medical Campus | [Colorado Multiple Institutional Review Board (COMIRB)](https://research.cuanschutz.edu/comirb) | 20 |
| 4 | UT Southwestern Medical Center, Children’s Health Dallas | WCG | 40 |
| 5 | University of Minnesota | WCG | 5 |
| 6 | Wake Forest School of Medicine | Wake Forest School of Medicine | 44 |
| 7 | Ann and Robert H. Lurie Children’s Hospital of Chicago | Ann and Robert H. Lurie Children’s Hospital of Chicago | 19 |
| 8 | Rutgers – New Jersey Medical School | WCG | 36 |

WCG = Western-Copernicus Group Institutional Review Board
