# Supplementary material for: BIS Guided Titration of Sevoflurane in Pediatric Patients Undergoing Elective Surgery: A Randomized Controlled Trial
Source: Paediatr Anaesth. 2025 Jan 4;35(4):277–86. doi: 10.1111/pan.15057 (PMC11883502; doi:10.1111/pan.15057)
Supplement: Supplementary file 2 — Table S2. [file PAN-35-277-s002.docx]

Supplementary Table 2: Comparison of End-Tidal Sevoflurane Concentration during Maintenance Phase by Treatment Arm (pre-planned, N=132)

| **Age Group** | **Statistic** | **BIS™ Guided**  **(n=56)** | **Control**  **(n=76)** | **p-value** |
| --- | --- | --- | --- | --- |
| 4 – 8 years | Mean±SD (n)  95% CI | 2.1±0.3 (19)  (1.7, 2.6) | 2.5±0.3 (24)  (1.7, 3.0) | 0.0008* |
| 9 – 12 years | Mean±SD (n)  95% CI | 1.7±0.5 (18)  (1.1, 2.5) | 2.2±0.6 (23)  (1.3, 3.5) | 0.008* |
| 13 – 18 years | Mean±SD (n)  95% CI | 1.5±0.3 (19)  (0.8, 2.0) | 1.9±0.5 (29)  (0.9, 2.7) | 0.007* |
| All | Mean±SD (n)  95% CI | 1.8±0.4 (56)  (0.8, 2.6) | 2.1±0.5 (76)  (0.9, 3.5) | <0.0001* |

SD, standard deviation; CI, confidence interval

Significance level set at p<0.05
