# Supplementary material for: BIS Guided Titration of Sevoflurane in Pediatric Patients Undergoing Elective Surgery: A Randomized Controlled Trial
Source: Paediatr Anaesth. 2025 Jan 4;35(4):277–86. doi: 10.1111/pan.15057 (PMC11883502; doi:10.1111/pan.15057)

**Supplementary Figure 1.** Regression Analysis End-Tidal Sevoflurane Concentration During Maintenance Phase by Age Group and Treatment Arm. The variables included in the main effects regression were end-tidal sevoflurane concentration, age group, and treatment group. The 9-12 years age group served as the reference group (reference is black dashed line). Individual data points are represented by blue circles for the 4-8 years group, red cross marks for the 9-12 years group, and green hatch marks for the 13-18 years group. Treatment group is represented on the x-axis, where 0 represents the control group and 1 represents the BIS™ guided group. The y-axis represents end-tidal sevoflurane concentration. The gray dotted line represents the regression line for the 4-8 years age group. The dark gray dotted line represents the regression line for the 9-12 years age group. The gray dashed-dotted line represents regression line for the 13-18 years group.


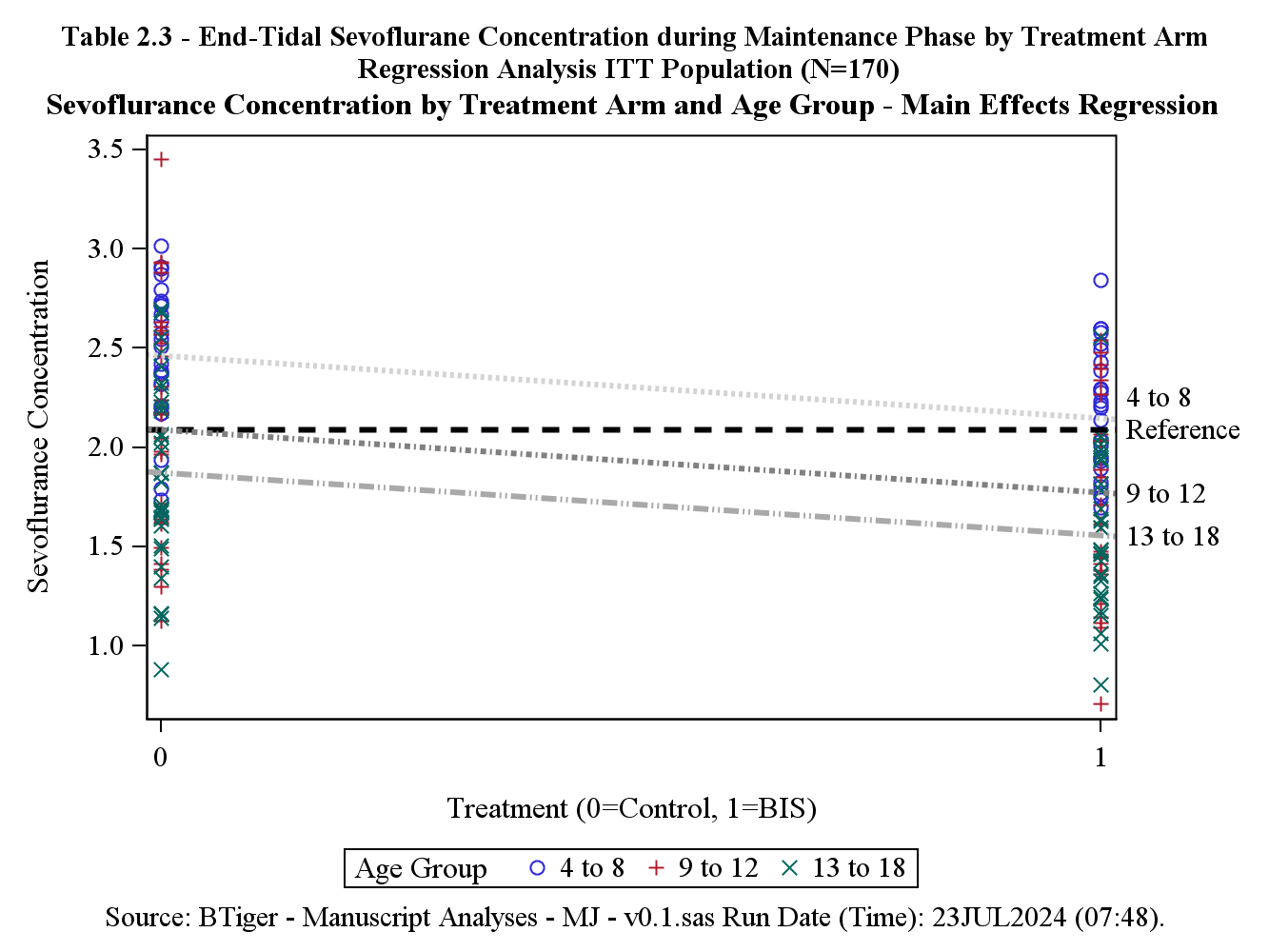

Supplement: Supplementary file 3 — Figure S1. [file PAN-35-277-s001.docx]
